# Transcriptional and Alternative Splicing Regulation of Autophagy and Vesicle Transport Pathways in Large Yellow Croaker Cells During Megalocytivirus Infection

**DOI:** 10.3390/ani16081259

**Published:** 2026-04-20

**Authors:** Zaiyu Zheng, Hongshu Chi, Xiaodong Liu, Xiuxia Chen, Ying Pan, Hui Gong

**Affiliations:** 1 Biotechnology Institute, Fujian Academy of Agricultural Sciences, Fuzhou 350003, China; cammy_zh@hotmail.com (Z.Z.); chihongshu@faas.cn (H.C.); xdliu777@163.com (X.L.); xiuxiachen@163.com (X.C.); py19860220@sina.com (Y.P.); 2 Fujian Provincial Engineering Technology Research Center for Aquatic Disease Control and Prevention, Fuzhou 350003, China; 3 State Key Laboratory of Mariculture Breeding, Ningde 352115, China

**Keywords:** non-lytic egress, host–virus interaction, RNA-seq analysis, alternative splicing regulation, authphagy, vesicle-mediated transport, aquatic virology

## Abstract

Infection of the large yellow croaker (*Larimichthys crocea*) embryo cell line YCE1 with megalocytivirus strain FD201807 leads to accumulation of capsid-deficient viral intermediates within intracellular vesicles at 48 h post-infection (a phenotype associated with non-lytic egress), which coincides with the initial peak of viral genomic copies. To characterize the host molecular response during this critical stage, we performed time-course RNA sequencing at 24, 48, 96, and 144 hpi. Integrated analysis identified 6661 differentially expressed genes (DEGs) and 1138 differential alternative splicing (DAS) events affecting 892 genes, with DAS event abundance peaking at 48 h. DAS genes in autophagy and Golgi vesicle transport pathways, both integral to animal innate immunity, were significantly enriched exclusively at this timepoint, featuring novel mutually exclusive exon (MXE) isoforms in *gopc* (Golgi-associated PDZ and coiled-coil motif containing) and *rint1* (RAD50 interactor 1). Weighted gene co-expression network analysis (WGCNA) of DEGs identified *mapk9* (mitogen-activated protein kinase 9) and *map1lc3a* (microtubule-associated protein 1 light chain 3 alpha) as hub genes within modules enriched for autophagy-related functions. Separate co-expression analysis of DAS genes revealed *rnf5*, *rimoc1*, and *golga4* as hub genes, with *gopc* exhibiting only a single linkage to *rnf5*. These findings implied concurrent transcriptional and virus-induced host splicing regulation of vesicle-associated innate defense pathways and suggest that splicing-derived features may serve as potential candidates for diagnostics or prevention against megalocytivirus disease in *L. crocea*.

## Simple Summary

Megalocytiviruses are large double-stranded DNA viruses in the family *Iridoviridae* that cause severe disease in farmed fish. In large yellow croaker, an important aquaculture species in China, these viruses lead to high-mortality outbreaks during warm summer months. A strain isolated from this fish exhibits a host-specific infection phenotype: in large yellow croaker cells, the virus generates incomplete viral intermediates that accumulate within intracellular vesicles, whereas in cells from other fish species it assembles mature virions. To investigate the host response associated with this distinct behavior, the study analyzed gene expression and splicing changes in infected large yellow croaker cells. Coordinated alterations were observed in genes governing Golgi vesicle transport and autophagy, two central pathways of the innate immune response. These molecular changes coincide with the presence of viral intermediates in vesicles, suggesting the virus may exploit the host’s vesicle trafficking system for non-lytic egress, potentially enabling persistent spread. These results provide new insights for developing diagnostic tools or antiviral strategies against viral diseases in this economically important species.

## 1. Introduction

Megalocytivirus is one of the more recently established genera within the family Iridoviridae, which comprises large double-stranded DNA viruses that infect over 180 fish species worldwide [1]. As the center of aquaculture industry in the world, East/Southeast Asia contributed the earliest and primary epidemiological foci for Megalocytivirus outbreaks [2]. Large yellow croaker (*Larimichthys crocea*) is one of the most popular aquaculture species in China, since the 2000s, the output of marine cage-cultured *L. crocea* in Fujian province has suffered from summer outbreaks of megalocytivirus, which have been marked by a high mortality rate of juveniles [3]. Mature megalocytivirus virions are non-enveloped, with hexagonal capsids, which can be detected in multiple tissues and organs of *L. crocea* including kidney, spleen, liver, brain, heart, gill, blood, yet their transmission pathways are still not verified [4,5,6].

To date, over 40 viruses across more than 20 families—spanning all known nucleic acid types—have been documented to transport via the host’s vesicle system; this non-lytic viral transmission mode, so-called vesicle-mediated en bloc transmission, has come into focus in the last two decades [7]. Unlike traditional single-virus-particle transmission, which is usually accompanied with cell lysis, the vesicle-hijack mechanism allows unassembled viral particles to cloak themselves as exosomes, autophagosomes, multivesicular bodies (MVB), or even apoptosis bodies [8], and initiate infection collectively through vesicle-mediated transport mechanisms such as endocytosis, exocytosis, efferocytosis, and plasma membrane fusion with enhanced infectivity [9]. Recent studies have indicated that vesicle-transported viral particles do not need complete capsids, and even mutated nucleic acids propagate successfully via population effects, so-called the “Trojan horse tactic” of multiple viral Quasispecies [10]. To help the viral cargo delivery via vesicles, viruses strategically employ the autophagy and Golgi transport pathways to enhance replication, acquire envelopes, and facilitate non-lytic release. Picornaviruses, Coronaviruses, Herpesviruses and Flaviviruses have all been reported to involve in the Golgi-ER (endoplasmic reticulum) autophagy crosstalk: they acquire membranes derived from the ER and Golgi to form double-membrane replication organelles and viral envelopes, and exploit Golgi-autophagosome hybrids for non-lytic egress. These processes are partially dependent on autophagy machinery components such as ATG proteins, LC3/LC3 II, SQSTM1 and GABARAP GTPases [11,12,13,14,15,16,17,18].

Most records of vesicle-mediated viral transmission have come from important human and mammalian pathogens, for example, poliovirus, rhinovirus, Coxsackievirus, hepatitis A/C virus (HAV/HCV), Ebola, Zika, West Nile virus (WNV), Enterovirus, human papillomavirus (HPV), Severe Acute Respiratory Syndrome Coronavirus 2 (SARS-CoV-2), African swine fever virus (ASFV) and bluetongue virus (BTV), but few examples involved aquatic viruses [16,19,20,21,22,23,24,25,26,27,28]. Recent studies on Singapore grouper iridovirus (SGIV) demonstrated that single mature SGIV particles could be transported individually within early/late endosomes (EE/LE) [29,30]. Previous studies utilizing the Yellow Croaker Embryonic type-I (YCE1) cell line revealed that infection with another RSIV sub-type megalocytivirus strain, large yellow croaker iridovirus CZ (LYCIV-CZ), resulted in the accumulation of capsid-deficient viral particles within cytoplasmic vesicles, double-membrane autophagosome-like structures, and multivesicular bodies (MVBs) [31]. Consistent with its high genetic homology to LYCIV-CZ and its proven capacity for efficient mature virion assembly in MFF-1 cells [6], FD201807 recapitulates this vesicle-related incomplete assembly phenotype in YCE1, a cell line derived from the natural host of the virus. This observation suggests that the assembly defect is a specific outcome of the interaction between this viral genotype and the natural host cellular environment, rather than an intrinsic replication deficiency.

Based on these observations, we hypothesized that the autophagy and Golgi vesicle transport pathways play pivotal roles in the viral life cycle within this specific host system. To comprehensively understand the regulatory mechanisms underlying these processes, analyses of the dual layers of the host response, encompassing both differential gene expression and differential alternative splicing, will be an effective strategy. This approach is particularly relevant given that host alternative splicing profiles of precursor messenger RNAs (pre-mRNAs) are extensively altered during infection, representing a deliberate strategy to bypass host immunity. Pathogens manipulate the splicing machinery via direct binding, exemplified by Influenza A nonstructural protein 1 (NS1) sequestering small nuclear RNA (snRNA) U6 and SARS-CoV-2 nonstructural protein 16 (NSP16) targeting snRNA U1 [32,33], or through indirect signaling cascades that modify splicing factor activity. Consequently, this reprogramming fosters a pro-viral state by dampening immune responses, such as through the generation of dominant-negative signal transducer and activator of transcription 1 (STAT1) isoforms [34,35], and by blocking cell death pathways [36], underscoring splicing regulation as a pivotal aspect of viral pathogenesis.

In the present study, to explore the vesicle-related intracellular mechanisms governing megalocytivirus infection in vitro, we performed a time-course comparative transcriptomic analysis of YCE1 cells infected with strain FD201807. The temporal expression profiles of differentially expressed genes (DEGs) and differential alternative splicing (DAS) events were characterized. Furthermore, weighted gene co-expression network analysis (WGCNA) [37] was employed to identify key genes and pathways involved in the host–pathogen interaction of megalocytivirus.

## 2. Materials and Methods

### 2.1. Virus Titration and Cell Infection

The YCE1 cells (passage 55) were inoculated into 75 cm^2^ culture flasks (Corning Inc., Corning, NY, USA), and incubated at 26 °C in Leibovitz’s L-15 medium (Basalmedia, Shanghai, China) supplemented with 15% fetal bovine serum (Oricell, Guangzhou, China) for 18 h, and then transferred into the maintenance medium (L-15 supplemented with 5% FBS) at 28 °C for 4 h before the infection. The megalocytivirus FD201807 strain, originally isolated from the kidney tissue of infected *L. crocea* collected from Ningde, Fujian province [6], was purified according to a modified method [4]. The viral titer was determined using the classical 50% tissue culture infective dose (TCID_50_) assay and calculated by the Reed–Muench method [38], and the virus was inoculated at a specific multiplicity of infection (MOI) based on this titer. Cell viability was measured via Trypan Blue staining. Viral replication was monitored by quantitative PCR.

### 2.2. Sample Preparation

The infected YCE1 cells were divided into three groups for electron microscopy (EM), major capsid protein (*mcp*) gene copies measurement (MC), and transcriptomic analysis (TR) respectively, each sample contained three repeats (*n* = 3), and equal flasks of uninfected cells were kept as the control group (Table 1). For the EM group, the infected cells were quick-fixed and harvested as described [31]. As for the MC and TR groups, after a single wash with 0.01 M PBS, 4 mL of FreeZol Reagent (Vazyme, Nanjing, China) was added per flask to fully cover the cell layer; finally, the cells were harvested with cell scrapers (Corning) gently. The MC samples were stored at −80 °C for quantitative real time polymerase chain reaction (qPCR), while the TR samples were transferred into cryovials and treated with liquid nitrogen.

### 2.3. Electron Microscopy

The harvested EM group cells were incubated at 4 °C overnight, ultra-thin sectioning and transmission electron microscopy (TEM) were subsequently carried out by Servicebio Technology Co., Ltd. (Wuhan, China).

### 2.4. Temporal Viral Load Measurement via qPCR Assay

The viral copy numbers in the infected YCE1 cells were quantified over a time course according to Table 1. For each sample, from 250 μL of the freeze-thawed infected cell suspension, the total DNA was extracted using a Tissue DNA kit (Vazyme), then eluted with 50 μL of Diethyl pyrocarbonate (DEPC)-treated water as PCR templates. One pair of primer designated as *Q-MCP F/R*, which were specific to megalocytivirus *mcp* gene (GenBank Accession No. OK149102.1), were prepared as reported [4] (Table 2). The new MCP-pMD-19T plasmid was constructed with a pMD-19T vector cloning kit (Takara Bio Inc., Kusatsu, Shiga, Japan) using the *Escherichia coli* (*E. coli*) DH5α expression system. The recombinant plasmids were diluted to 10^10^–10^1^ copies·μL^−1^ to plot standard curves based on the Cycle threshold (Ct) values, by which the viral copy number was calculated [31]. The qPCR amplification of the specific segment of 105 bp was carried out via a QuantStudio^®^ 3 real-time PCR system (Thermo Fisher Scientific, Waltham, MA, USA) using a TB Green^®^ Premix Ex Taq™ II kit (Takara): the 20 μL reaction system contained 10 μL of TB Green^®^ Premix Ex Taq™ II (Tli RNaseH Plus) (Takara), 0.8 μL of each primer, 0.4 μL of ROX Reference Dye (10×) (Takara), 2 μL of cDNA template and ddH_2_O. The cycling conditions were: 95 °C for 30 s for pre-denaturation, followed by 40 cycles of 95 °C for 5 s, 60 °C for 34 s. The melt curve analysis conditions were: 95 °C for 15 s, 60 °C for 1 min, followed by 95 °C for 15 s. The viral copy numbers at different time points were calculated using the 2^−ΔΔCt^ method, each experiment was repeated at least three times. The data are shown as mean ± standard deviation (SD), and statistical significance was determined via multiple comparisons within the framework of one-way analysis of variance (Repeated Measures ANOVA, Turkey’s HSD test) using GraphPad Prism 9.5 (GraphPad Software, San Diego, CA, USA; www.graphpad.com, accessed on 31 July 2024).

### 2.5. High-Throughput Sequencing and Data Assembly

Construction of the library and high-throughput sequencing were conducted by Wuhan SeqHealth Tech Co., Ltd. (Wuhan, China) using a MGISEQ-T7 system (MGI Tech, Shenzhen, China). To get clean reads, the raw reads were filtered using the fastp software ver. 0.23.0 [39] (https://github.com/OpenGene/fastp, accessed on 6 July 2023) by removing the adapter sequences, the reads shorter than 15 bp, the reads containing bases with low quality scores (*Q* ≤ 20) exceeded 8%, and reads containing more than five ambiguous bases (N). Clean reads with similar UID sequences were merged using the kcUID processing software (SeqHealth Tech), enabling error correction and duplicate removal. The clean reads were mapped to the *L. crocea* genome (GWH Accession = GWHAATE00000000). The expression abundance and variations of the mapped reads were quantified using the RPKM (Reads per Kilobase per Million Reads) method [40], following the formula as below:

(1) $$\mathrm{RPKM}\;=\;\frac{\mathrm{total}\;\mathrm{exon}\;\mathrm{reads}}{\mathrm{mapped}\;\mathrm{reads}\;\left(\mathrm{millions}\right)\;\times \;\mathrm{exon}\;\mathrm{length}\;\left(\mathrm{KB}\right)}$$

The hierarchical clustering correlation heatmap and principal component analysis (PCA) correlation plot were generated by DESeq2 software ver. 1.38.3 (ρ > 0.8) [41] (https://bioconductor.org/packages/3.16/bioc/html/DESeq2.html, accessed on 15 August 2023).

### 2.6. Temporal Analysis of the Differentially Expressed Genes (DEGs) and the Differential Alternative Splicing (DAS) Genes

Differential expression analysis was carried out by DESeq2 between each two different groups. The genes with absolute logFC (log-fold change) > 1 and *p* < 0.05 were considered as differentially expressed genes (DEGs). The alternative splice events between different samples were classified and counted using the replicate Multivariate Analysis of Transcript Splicing (rMATS) software ver. turbo 4.1.2. [42] (https://github.com/Xinglab/rmats-turbo/releases/tag/v4.1.2, accessed on 18 December 2023). The analysis incorporated three biological replicates per group using a hierarchical model. This model employs a binomial distribution to account for read sampling variation and a logit-normal distribution to model variability across replicates. A likelihood-ratio test was used to assess significance. Key parameters included --libType fr-secondstrand, --readLength 131, and --cstat 0.0001. The junction reads and the reads on targets were calculated following the formula as below (where *ψ* means the exon inclusion levels, *I* represents the exon inclusion reads, *S* represents the exon skipping reads, *L_I_* means the length of inclusion form, and *L_S_* represents the length of skipping form):

(2) $$\hat{\psi }=\frac{I/L_{I}}{I/L_{I}+S/L_{S}}$$

And the splicing event significance thresholds were: *p* < 0.05, |Δ*ψ*| > 0.1.

### 2.7. Functional Enrichment and Clustering

The DEGs and DAS genes were further annotated by the gene ontology (GO) [43] (http://geneontology.org, accessed on 20 December 2023) and the Kyoto encyclopedia of genes and genomes (KEGG) [44] (http://www.genome.jp/kegg/, accessed on 20 December 2023) pathway enrichment analyses, respectively. After multiple test correction, the clustered profiles with *p* < 0.05 were considered to be significantly enriched. The grouped bubble plot of GO/KEGG-enriched genes in target terms/pathways was generated using the web-based Omicshare Tools (https://www.omicshare.com/tools/, accessed on 2 November 2025) [45].

### 2.8. Validation of Post-Infection Expression Patterns of the DEGs Using Quantitative Reverse Transcription-PCR (qRT-PCR)

The temporal expression profiles of four DEGs were quantified by qRT-PCR to validate the transcriptomic data. Based on a threshold of mean RPKM > 200, three genes, *psap* (prosaposin, GenBank accession no. XM_027276328.1), *rpl18a* (ribosomal protein L18a, GenBank accession no. XM_027272485.1), and the uncharacterized locus *LOC104920700* (predicted protein, GenBank accession no. XM_010733046.3), were selected to represent low (~400 RPKM), medium (~600 RPKM), and high (>1000 RPKM) expression levels. Additionally, the key autophagy-related gene *map1lc3a* (microtubule-associated protein 1 light chain 3 alpha, GenBank accession no. XM_027279266.1) was included for validation. The qRT-PCR primers were designed and synthesized by Sangon Biotech Co., Ltd. (Shanghai, China), and their sequences are listed in Table 2. The infected cell samples were treated with a FastPure^®^ cell/Tissue Total RNA Isolation Kit V2 (Takara), and the reverse transcription was conducted in the following steps: step 1, the RNA-Master Mix solution I was prepared (10 μL containing 2.0 μL of 5 × gDNA Eraser Buffer, 1.0 μL of gDNA Eraser, total RNA up to 1 μg, and RNase Free ddH_2_O) and incubated at 42 °C for 2 min; step 2, Master Mix solution II (10 μL containing 1.0 μL of PrimerScript RT Enzyme Mix l, 1.0 μL of RT Primer Mix, 4.0 μL of 5 × PrimeScript Buffer 2, and 4.0 μL of RNase Free ddH_2_O) was added to the reaction mixture of step 1; step 3, the total reaction system was kept at 37 °C for 15 min, then at 85 °C for 5 s. Subsequently, the qRT-PCR was carried out with a QuantStudio^®^ 3 real-time PCR system (Thermo Fisher Scientific) using TB Green^®^ Premix Ex Tag^TM^ II (Takara) in a 20 μL reaction system as described in Section 2.4.

### 2.9. Weighted Gene Coexpression Network Analysis (WGCNA)

To further describe the post-infection behavior of FD201807, and detect significant genes and pathways associated with its vesicle-related intracellular pattern, WGCNA R package ver. 1.74 (https://bioconductor.org/packages/3.20/bioc/html/WGCNA.html, accessed on 5 September 2025) [37] was used to analyze the sequencing data of both the DEGs and the DAS genes; the results were visualized using Cytoscape ver. 3.10.3 (http://cytoscape.org, accessed on 5 July 2025) and rMATSsashimiplot 2 pack (https://github.com/Xinglab/rmats2sashimiplot, accessed on 11 December 2025).

### 2.10. Expression Trend Analysis

The temporal expression trends of 13 significant genes identified by the GO and KEGG enrichment analyses were visualized via short time-series expression miner (STEM) [46] software on the OEbiotech Cloud platform (https://cloud.oebiotech.cn/, accessed on 4 February 2026), the expression trends of two long-term DEGs (genes differentially expressed at all sampling time points) were also analyzed as references (Table S1).

## 3. Results

### 3.1. Megalocytivirus Titration and Cell Infection

**Figure 1:**
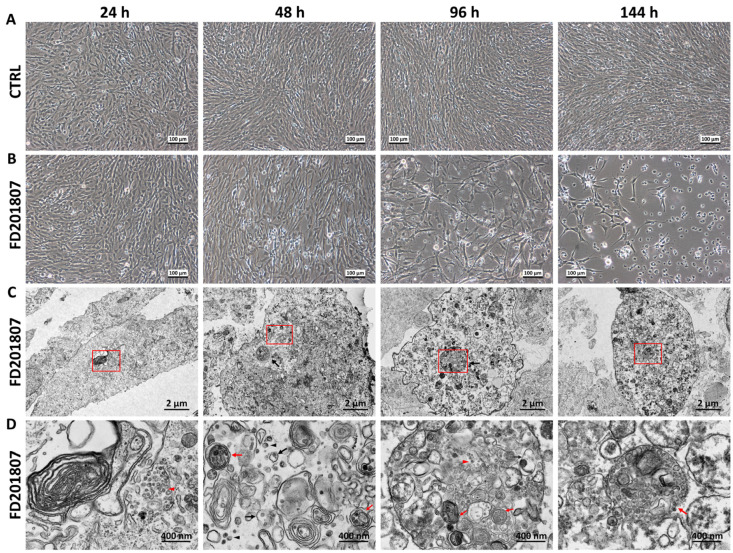
The temporal microscopy of passage 55 Yellow Croaker Embryonic type-I (YCE1) cells infected by the megalocytivirus strain FD201807. (**A**) Control group, 24–144 h in vitro. (**B**) Passage 55 YCE1 cells, FD201807 strain infected, 24–144 h following inoculation. (**C**) Transmission electron microscopy of the intracellular pattern of YCE1 cells, 24–144 h post FD201807 infection, N, nucleus; HITACHI HT7700, 3000×; scale bar = 2 μm. (**D**) The magnified view of the red-boxed region in the panel (**C**), showing the immature viral particles and vesicular structures: black triangles, dispersed viral particles; red triangles, clustered viral particles; small black arrows, single-membrane vesicles; small red arrows, double-membrane autophagosome-like organelles; big black arrow, multivesicular body (MVB); big red arrow, virions gathered tightly in inclusion; HITACHI HT7700, 20,000×; scale bar = 400 nm.

The tested megalocytivirus strain, FD201807, which was named after the location and date it was collected, could cause cytopathic effects (CPEs) on the YCE1 cells. The viral TCID_50_ was measured to be 10^−8.286^ × 100 μL^−1^, and the conventional MOI was determined to be 1.0. At 24 h post-infection, both the control cells and the infected cells were kept confluent under a phase contrast microscope (Figure 1A,B), while the transmission electron microscopy (TEM) revealed immature viral particles clustering in the cytoplasm, with no significant disruption of intracellular membranes or organelle structures observed (Figure 1C,D). At 48 hpi, significant CPEs were observed in the FD201807-infected group (Figure 1A,B). Under the TEM observation, the intracellular architecture remained largely intact, but exhibited cytoplasmic vacuolization. The vacuolated regions contained numerous immature viral particles: dispersed or encapsulated in cytoplasmic vesicles, autophagosome-like organelles, and multivesicular bodies (MVBs) (Figure 1C,D). At 72 hpi, the CPEs aggravated (Figure 1A,B), while the TEM photos showed that the infected cells contained disrupted organelles, and large, multilayered vesicular complexes derived from diverse virus-containing vesicles (Figure 1C,D). At 144 hpi, the infected group exhibited extensive cell detachment, and the cell viability was measured to be approximately 40%. The TEM images demonstrated abundant vacuolar structures, with immature virions gathered within. (Figure 1C,D). Almost all these virions detected were non-enveloped and capsid-deficient.

**Figure 2:**
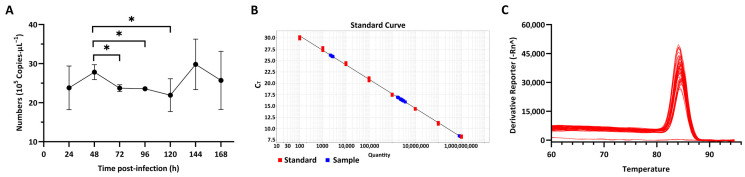
Viral load quantification. (**A**) Temporal dynamics of FD201807 major capsid protein (*mcp*) gene copies expressed in YCE1 cells, the values are displayed as mean ± SD (* *p* < 0.1, *n* = 3). (**B**) Standard curve for absolute quantification (*R*^2^ = 0.998). (**C**) Melting curve analysis showing primer specificity.

The viral genome was authenticated via qRT-PCR targeting the characteristic fragment of the *mcp* gene of megalocytivirus (GenBank Accession No. KY765672.1), yielding an identical 105 bp product. The copy number of FD201807 *mcp* peaked initially at 48 h post-infection (hpi), gradually declined to a nadir at 120 hpi, and surged to a second peak at 144 hpi before resuming a downward trend (Figure 2A). The standard curve exhibited excellent linearity (*R*^2^ = 0.998) with the regression equation *y* = −3.186*x* + 36.8096, 2 ≤ *x* ≤ 10 (Figure 2B), and a single peak in the melting curve confirmed amplification specificity (Figure 2C).

### 3.2. High-Throughput Sequencing and Data Qualification

**Figure 3:**
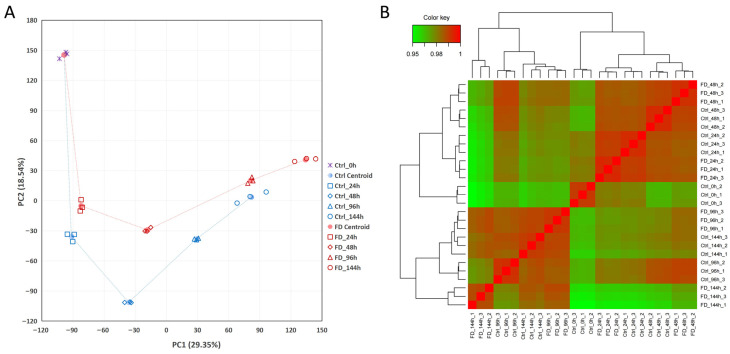
The inter-sample correlation analysis of the FD201807-infected YCE1 replicates. (**A**) Principal component analysis (PCA) correlation plot of the full transcriptional profiles. (**B**) Hierarchical clustering correlation heatmap of all groups.

The PCA results showed no significant outlier or isolated samples, demonstrating the stability of the sample quality. Along PC1 (29.35%) and PC2 (18.54%), the intra-group repeatability and inter-group separation indicated the distinct patterns of gene expression patterns and the significant impact of processing factors (Figure 3A). In the hierarchical clustering analysis, the correlation coefficient between samples in the same group was mostly 0.98–1.0 (red to dark red), while the correlation coefficient between inter-group samples was mostly 0.95–0.98 (green to yellow), and the proportion of red area was overwhelmingly higher than that of the green areas (Figure 3B). The heat map revealed that all biological repeat samples were clustered in order by treatment group and time point without cross-group mixing, which further verified the consistency of gene expression patterns of the FD201807-infected YCE1 cells.

### 3.3. Validation of the RNA-Seq Using qRT-PCR

In order to validate the RNA-seq results, the expression level of four DEGs, *psap*, *rpl18a*, *LOC104920700*, and *map1lc3a*, were quantified with qRT-PCR analysis. The results showed that the temporal expression patterns of the 4 DEGs were comparable to those graphed by RNA-seq (Figure S1), thus the RNA-seq results were verified.

### 3.4. The Temporal Pattern of Differentially Expressed Genes (DEGs) and Differentially Alternative Splicing (DAS) Events Induced by FD201807 Infection

**Figure 4:**
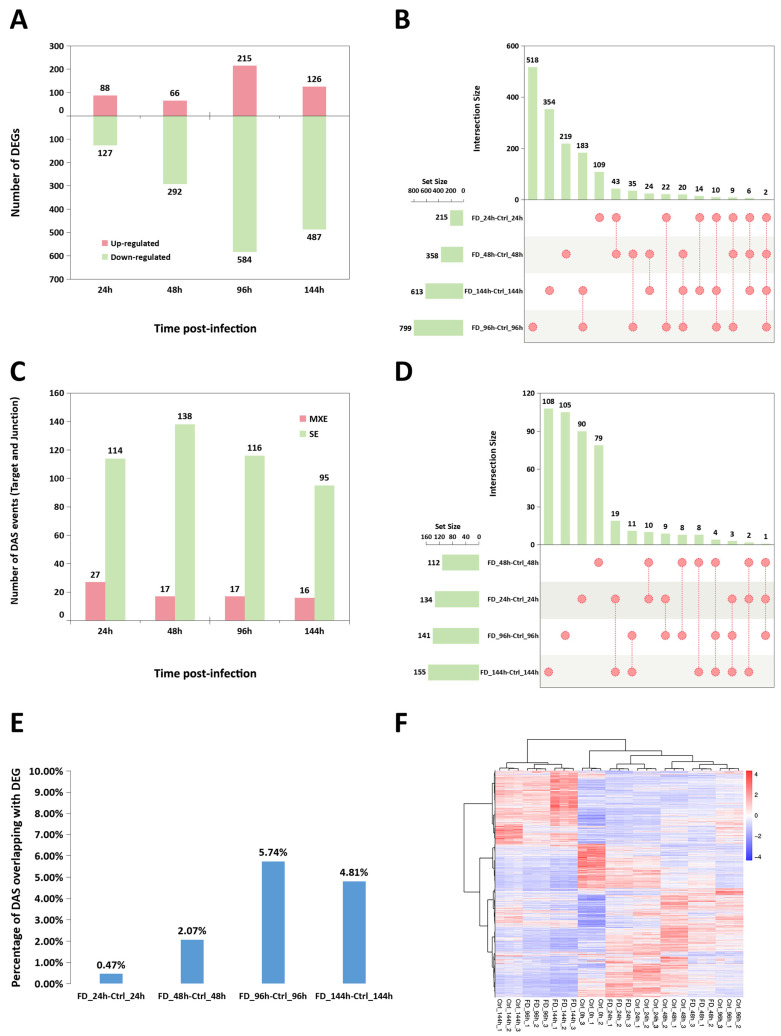
The temporal pattern of differentially expressed genes (DEGs) and differential alternative splicing (DAS) genes. (**A**) Number of DEGs at 24, 48, 96 and 144 h post-infection. (**B**) The UpSet plot of DEG overlaps. (**C**) Number of DAS genes at 24, 48, 96 and 144 h post-infection. (**D**) The UpSet plot of DAS gene overlaps. (**E**) The overlap ratio between DEGs and DAS genes at each sampling time point. (**F**) Heatmap visualization of global gene expression patterns based on hierarchical clustering (*p* < 0.05, |Log2FC| > 1), relative expression levels of each transcript (rows) in each sample (column) were shown. Color scale: red (up-regulated), blue (down-regulated), red or blue intensity indicated the regulation magnitude. Ctrl_24h_1: Control group, sampled at 24 h after being transferred into the same maintenance medium as the infected group, Parallel sample No. 1; FD_24h_1: FD201807-infected group, sampled at 24 h post-infection, Parallel sample No. 1, and so on. The same applies below.

Multiple comparison analysis were conducted to further investigate the temporal changes in gene expression profiles of the infected cells. The comparisons across total 20 groups identified 6661 differentially expressed genes (DEGs) and 1138 differential alternative splicing (DAS) events involving 892 genes. When compared to the uninfected samples, at 24, 48, 96 and 144 hpi, 215, 358, 799, and 615 DEGs were detected in FD201807-infected YCE1 cells respectively; at each time point, the up-regulated genes were significantly outnumbered by the down-regulated ones, which accounted for 73.29 ± 10.14% (mean ± SD, *n* = 4) of the total DEGs (Figure 4A). The shared DEGs among distinct groups were graphically represented through an UpSet Plot (Figure 4B). At 24, 48, 96 and 144 hpi, 141, 155, 133 and 111 DAS events were recorded versus the control groups (Figure 4C). Only mutually exclusive exon (MXE) and skipped exon (SE) alternative splicing events were detected, and the temporal analysis revealed that SE events consistently accounted for >80% of total DAS events at all measured time points (mean ± SD: 85.67 ± 3.51%, *n* = 4). The UpSet Plot of shared DAS genes among distinct groups was presented as well (Figure 4D). A low overlap was observed between DEGs and DAS genes across all sampling time points, reaching a maximum of only 5.74% (Figure 4E). A hierarchical clustering heatmap of all DEGs was plotted based on the RPKM value (Figure 4F).

### 3.5. GO and KEGG Enrichment Analysis for the DEGs and the DAS Genes

**Figure 5:**
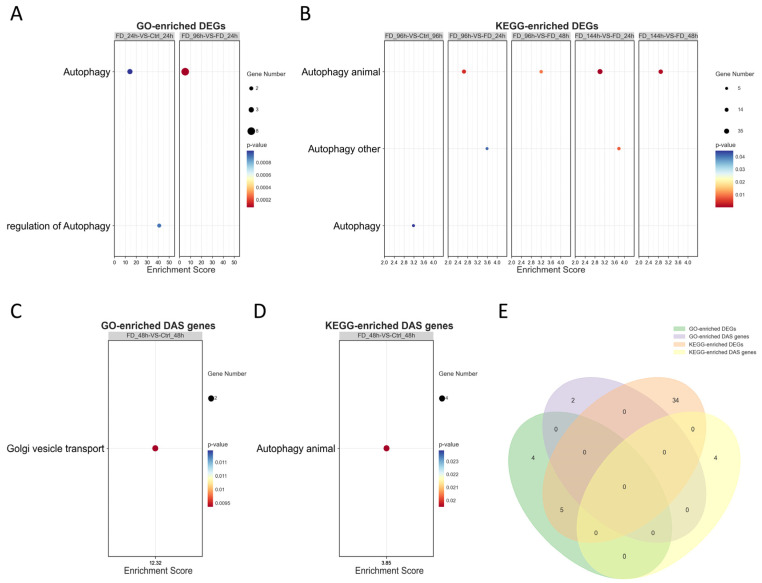
Gene Ontology (GO) and Kyoto Encyclopedia of Genes and Genomes (KEGG) enrichment analyses of DEGs and DAS genes associated with autophagy and Golgi vesicle transport. (**A**) GO enrichment results for DEGs. (**B**) KEGG pathway enrichment results for DEGs. (**C**) GO enrichment results for DAS genes. (**D**) KEGG pathway enrichment results for DAS genes. (**E**) Venn diagram showing the overlap of all analyzed transcripts. In the bubble plots (**A**–**D**), the bubble size represents the number of enriched genes, and the color gradient indicates the statistical significance (*p*-value).

To explore the vesicle encapsulation of FD201807 particles, we performed GO and KEGG enrichment analyses on the identified DEGs and DAS genes. The analyses identified seven and 32 autophagy-associated DEGs, respectively, with three common genes (Table S2). Specifically, two biological process terms (Autophagy [GO:0006914] and Regulation of autophagy [GO:0010506]) were significantly enriched (Figure 5A). In the KEGG analysis, three pathway entries were annotated: autophagy and autophagy-animal (both mapped to lco04140), and autophagy-other (lco04136) (Figure 5B). GO analysis revealed that two DAS genes were significantly enriched in Golgi vesicle transport (GO:0048193; Figure 5C), while four DAS genes were associated with autophagy in the KEGG analysis (Figure 5D). A Venn diagram illustrating the overlap among these five enriched terms detected five differentially expressed transcripts derived from three unique genes common to the selected methods (Figure 5E). Notably, all these transcripts were identified at 48 hpi compared to the control (Figure 5C–E; Table S3).

**Figure 6:**
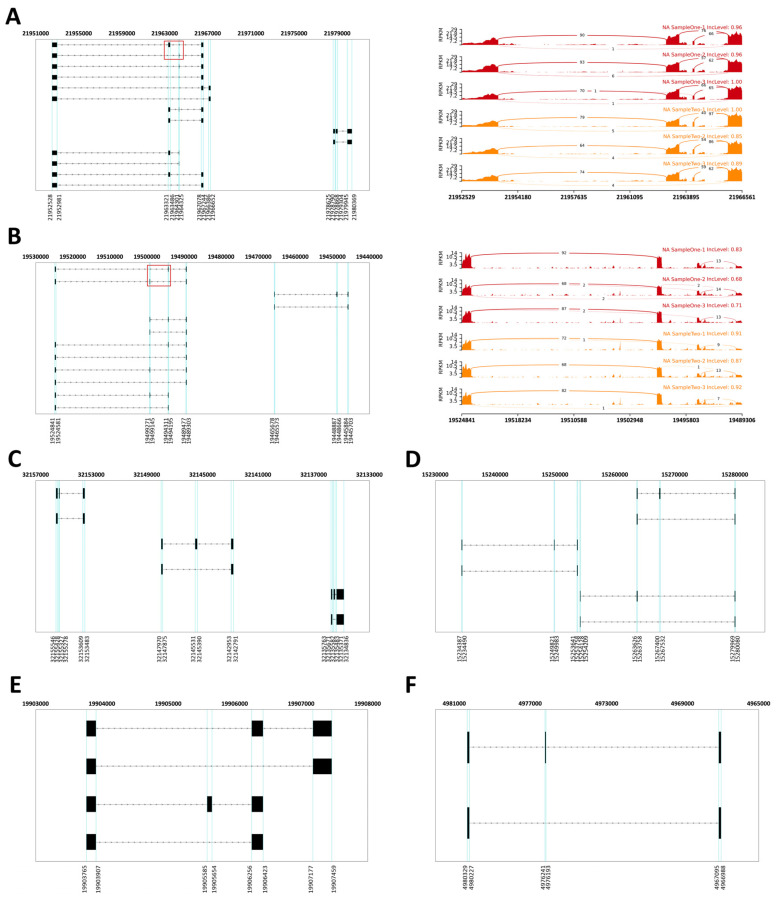
Quantitative visualization of six candidate DAS genes at 48 h post-FD201807 infection. The schematics summarize splicing events of (**A**) *gopc*, (**B**) *rint1*, (**C**) *tsc2*, (**D**) *vmp1*, (**E**) *pten*, and (**F**) *nt5c2b*. Mutually exclusive exon (MXE) splicing events are highlighted in red boxes, and exon boundaries are labeled using blue vertical strings and genomic coordinates, with exons as black boxes, introns as lines, and transcriptional direction indicated by arrows. Sashimi plots comparing MXE splicing patterns between control (red) and FD201807-infected (yellow) groups are presented in the right panels of (**A**,**B**). The *x*-axis represents genomic coordinates, the *y*-axis shows normalized RPKM values. Arc heights correspond to splice junction read counts (labeled numerically), and IncLevel indicates the normalized exon inclusion ratio. Full gene descriptions are listed in Table S1. Sashimi plots for all skipped exon (SE) events are shown in Figure S3.

Regarding splicing patterns, the Golgi-related genes *gopc* (golgi-associated PDZ and coiled-coil motif-containing) and *rint1* (RAD50 interactor 1) exhibited both MXE and SE events (Figure 6A,B). In contrast, the four autophagy-related genes *tsc2* (tuberous sclerosis complex 2), *vmp1* (vacuole membrane protein 1), *pten* (phosphatase and tensin homolog), and *nt5c2b* (5′-nucleotidase, cytosolic IIb) displayed only SE events (Figure 6C–F). Detailed structural diagrams of all splice variants for these DAS genes are provided in Figure 6.

### 3.6. STEM Analysis

The short time-series expression miner (STEM) analysis presented the temporal expression trends of the predicted important functional genes throughout the infection process and revealed a significant long-term up-regulation of eight out of fifteen transcripts in total. The two long-term DEGs, Atypical chemokine receptor 3 (*LOC104934108*) and Fras1-related extracellular matrix 1b (*frem1b*), did not show significant clustering trends in long-term expression after FD201807 infection. With the exception of *rint1*, the remaining five DAS genes (*gopc*, *pten*, *tsc2*, *nt5c2b* and *vamp1*) also showed no significant trends (Table S1 and Figure S2).

### 3.7. WGCNA

**Figure 7:**
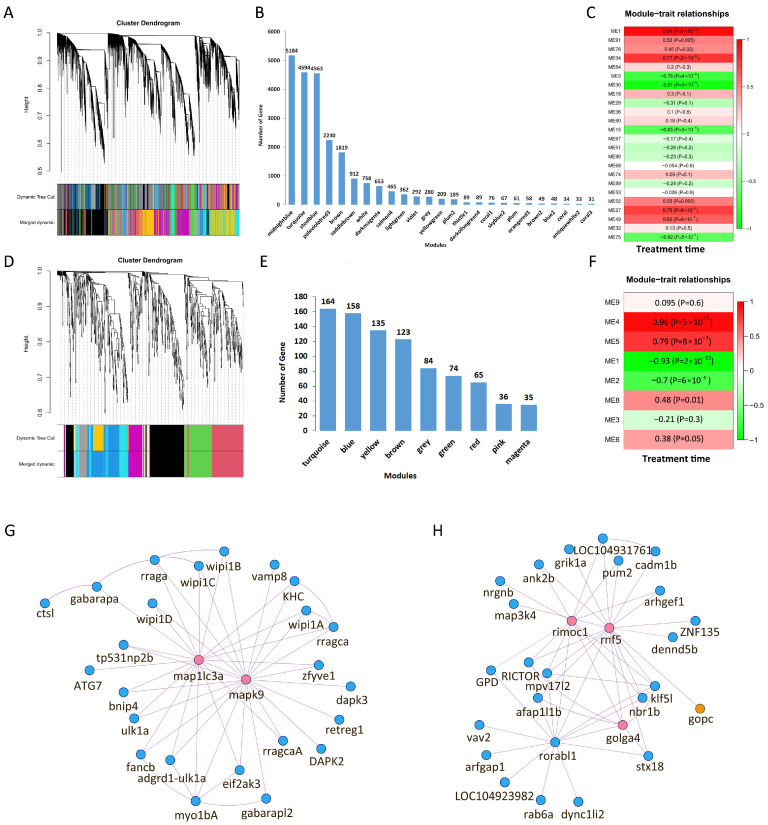
Weighted gene co-expression network analysis (WGCNA) of DEGs and differentially alternative splicing DAS genes. (**A**) Hierarchical clustering dendrogram of DEGs based on average linkage clustering. Modules were identified using the dynamic tree cut algorithm and assigned distinct colors. (**B**) Bar plot showing the number of DEGs distributed across the 25 identified modules. (**C**) Heatmap of module–trait relationships between DEG modules and post-infection time points. (**D**) Hierarchical clustering dendrogram of DAS genes. (**E**) Bar plot showing the number of DAS genes distributed across the nine identified modules. (**F**) Heatmap of module–trait relationships between DAS gene modules and post-infection time points. In panels (**C**,**F**), green and red colors indicate negative and positive correlations, respectively. (**G**) Co-expression network of autophagy-related DEGs within Module 1 (corresponding to the turquoise cluster in panel (**A**)). (**H**) Co-expression network of Golgi vesicle transport-associated DAS genes within Module 5 (corresponding to the green cluster in panel (**D**)). In the networks, hub genes are highlighted in pink, and the singly connected key gene in panel (**H**) is highlighted in yellow. A full list of network members and the specific identification of hub and enriched genes is presented in Table S4.

To identify gene clusters associated with treatment progression, WGCNA was performed. For gene expression, 23,156 genes were categorized into 25 co-expression modules (Figure 7A,B). Module 1 (turquoise) exhibited the strongest positive correlation with treatment time (*r* = 0.94, *p* = 6 × 10^−13^), indicating that these genes are tightly associated with the temporal response to the treatment (Figure 7C). Regarding alternative splicing (AS), 875 differential AS events were allocated into nine modules (Figure 7D,E). The module–trait analysis revealed that both Module 4 (yellow) (*r* = 0.96, *p* = 5 × 10^−15^) and Module 5 (green) (r = 0.79, *p* = 8 × 10^−7^) showed significant positive correlations with treatment time (Figure 7F). These modules were therefore prioritized for further investigation into splicing regulation.

### 3.8. Co-Expression Network Construction

In the GO and KEGG enrichment analyses, all significantly enriched DEGs were mapped to Module 1 of the DEG network, including multiple variants of WD repeat domain, phosphoinositide interacting 1 (*wipi1*), ras-related GTP binding Ca (*rragca*), and napsin A aspartic peptidase (*napsa*). The enriched DAS genes were identified in Modules 4 and 5 of the DAS network. We calculated the module membership (kME) values for these enriched genes (Tables S1 and S2) and selected those with kME > 0.8 for network construction (Figure 7G,H). Among the autophagy-related DEGs, *map1lc3a* and mitogen-activated protein kinase 9 (*mapk9*, also known as *jnk2*) were identified as hub genes (Figure 7G). In the DAS gene network, hub genes included ring finger protein 5 (*rnf5*), rab7a interacting mon1-ccz1 complex subunit 1 (*rimoc1*), and golgin A4 (*golga4*). Notably, *gopc* was connected only to *rnf5*; it was the sole representative included in the network out of the six initially enriched DAS genes (Figure 7H). Detailed transcript IDs and annotations for all network genes are available in Table S4.

## 4. Discussion

The classical dichotomy between enveloped and non-enveloped viruses is increasingly being challenged by evidence that non-lytic release is not exclusive to enveloped viruses. Our study contributes to this evolving understanding by demonstrating that capsid-deficient particles accumulate within vesicular structures in YCE1 cells infected by the megalocytivirus strain FD201807. This phenomenon, consistent with our prior observations of the LYCIV-CZ strain [31], supports the hypothesis that non-enveloped viruses can exploit membrane-trafficking pathways [47,48,49] as a specific adaptation to their natural hosts.

In mammalian systems, non-lytic egress is well-documented, where viruses such as poliovirus, coxsackievirus, rhinovirus, and hepatitis A and E viruses utilize various membrane-trafficking mechanisms to achieve non-lytic transportation. These mechanisms include biosynthetic secretion via the ER and Golgi apparatus [50,51,52], endo/lyso/autophagosomal exocytosis via exosome, autophagosome, lysosome, and multivesicular body, and plasma membrane budding [16,49,53]. However, despite these advances, the corresponding mechanisms in aquatic viruses represent a significant knowledge gap [16,49]. We propose that FD201807 may interact with host vesicle systems, potentially via autophagy or Golgi transport, based on the enrichment of related pathways identified in our transcriptomic and WGCNA analyses.

The vesicle-encapsulation of megalocytivirus assembly intermediates within host cells and the significant CPEs indicated that the immature virions can keep their infectivity while transporting via vesicular pathways. The virus strain FD201807 belongs to *Megalocytivirus pagrus 1*, the red seabream iridovirus (RSIV) genotype, typically exhibiting non-enveloped, dispersed cytoplasmic distribution or subcrystalline-like arrangement with intact hexagonal capsids [6]. In previous studies, FD201807 strain showed distinct intracellular distribution patterns when infecting different cell lines: in the mandarin fish fry cell line MFF-1, viral particles were dispersed throughout the cytoplasm with intact capsids [6], whereas in *L. crocea* embryo cell line YCE1, they clustered as capsid-deficient assembly intermediates within various vesicular structures (Figure 1D). This clustering pattern was similar to another RSIV-subtype megalocytivirus isolate LYCIV-CZ [31], suggesting a feature that may exhibit host specificity under similar multiplicity of infection (MOI) and infection duration. The TCID_50_ test revealed that FD201807’s titer in YCE1 cells was comparable to other iridoviruses in common cell models, indicating the full infectivity of these vesicle-encapsulated assembly intermediates [31,54,55,56,57,58,59]. Since recent advances in viral en bloc transmission demonstrate that non-lytic egress via extracellular vesicles (EVs) allows multiple viral genomes to form a collective infectious unit, bypassing the limitations of single-particle infectivity, this morphological disparity suggests that the production of diverse, non-canonical particles in natural host cells is not an assembly defect, but rather a regulated strategy of collective transmission [31]. We hypothesize that this heterogeneity constitutes a viral quasispecies swarm, where capsid-deficient virions undergo functional complementation [10]. By releasing a spectrum of genomic information via various vesicles, the virus ensures a high multiplicity of infection, effectively converting a heterogeneous particle population into a cooperative unit for efficient propagation.

To investigate the host factors underlying the atypical post-infection intracellular pattern of FD201807, we analyzed transcriptional and post-transcriptional responses in YCE1 cells. Although global gene expression was progressively suppressed, with down-regulated differentially expressed genes (DEGs) dominating over time, key components of autophagy and Golgi vesicle transport pathways exhibited a distinct biphasic pattern: transient early suppression followed by sustained upregulation that coincided with significant cytopathic effects (CPEs) in the late stage of infection. (Figure 1, Figure 2 and Figure 5A,B and Figure S2, Table S2). WGCNA for the DEGs identified *map1lc3a* (Encoding LC3A) and *mapk9* as hub genes of the co-expressional network, both continuously up-regulated throughout infection (Figure 7A and Figure S2). The MAPK (mitogen-activated protein kinase) gene family is reported to take part in the regulation of host cell apoptosis and viral replication during aquatic viral infections: for instance, inhibition of the ERK/p38 MAPK pathway significantly affects red-spotted grouper nervous necrosis virus (RGNNV)-induced CPEs, and the JNK downstream effector c-Jun plays essential roles in SGIV assembly and replication [60,61,62]. Meanwhile, *maplc3a* plays dual roles in host–virus interactions: canonical degradative autophagy and secretory autophagy along with its partners such as Mechanistic Target of Rapamycin (*mtor*), Unc-51-Like Autophagy-Activating Kinase (*ulk*), *Beclin-1* and Vacuolar Protein Sorting 34 (*vps34*) [63]. Generally the LC3A protein facilitates autophagosome elongation and maturation, and mediates viral degradation [63,64,65,66,67]; however, it also supports non-lytic egress of variant viruses via secretory autophagosomes or chimeric secretory autophagosome-MVB fusion organelles, a mechanism that may, in some cases, enable the co-packaging and collective transmission of viral quasispecies [10]. Examples of viruses using this route include poliovirus, coxsackievirus B, picornavirus and Zika [18,19,68,69,70,71]. Although this secretory autophagy-mediated egress is well documented for numerous RNA viruses, only a few DNA viruses have been reported to exploit this non-lytic mechanism: for example, the Human cytomegalovirus (HCMV) and Acanthamoeba large marseillevirus (*Marseillevirus marseillevirus*) [72,73]. Intriguingly, another megalocytivirus, infectious spleen and kidney necrosis (ISKNV), has been recently reported to activate host autophagy in grunt fin (GF-1) cells through the AMPK/mTOR/ULK1 and Beclin-1 (BECN1)/p62 (SQSTM1) pathways [12,74,75]. Together, these observations suggest that *map1lc3a* and *mapk9* may serve versatile roles in the host–pathogen interplay of aquatic DNA viruses. Given that FD201807 infection culminates in pronounced CPEs and efficient cell-to-cell spread, the persistent activation of these pathways and the concomitant accumulation of incomplete viral intermediates within vesicles likely represent a functional preparatory stage for the coordinated trafficking of heterogeneous viral genomes through host-derived membrane compartments.

Since the 1970s, most studies on precursor messenger RNA splicing in viral infection were focused on the splicing of viral genes, only in the recent decade, a growing interest has been motivating research on the host splicing changes during viral infection, and the consequent effects on host-viral interplay [76]. The virus-induced splicing changes can inhibit cellular immune signaling, prevent cellular apoptosis, or even try to create a more proviral cellular environment [77,78,79,80]. It is noteworthy that there is a significant overlap between mammalian viruses documented to alter the host splicing and those that utilize non-lytic vesicular transport, such as Human/Simian Immunodeficiency Virus (HIV/SIV), SARS-CoV-2, Zika, Dengue Virus (DENV), Porcine Reproductive and Respiratory Syndrome Virus (PRRSV), HCV [33,49,81,82,83,84,85,86,87,88,89], suggesting that the viral strategy of hijacking host splicing may be functionally dependent on subtle and non-destructive cellular exit mechanisms [76].

In our temporal transcriptomic analysis, alternative splicing activity peaked at 48 hpi (Figure 4C), in this critical time window, the intracellular copy number of FD201807 genome reached its first peak, but the CPEs were not significant (Figure 2), and the capsid-deficient viral particles were observed clustering in various vesicles (Figure 1D). Notably, DAS genes associated with Golgi vesicle transport (*gopc*, *rint1*) or autophagy (*pten*, *tsc2*, *vmp1*, *nt5c2b*) were significantly enriched only at 48 hpi; these pathways were absent in the functional enrichment results at any other time point. (Figure 5C,D and Table S3). Throughout the entire infection process of FD201807, the overlap between DEGs and DAS genes remained at a very low level (Figure 4E). Moreover, genes associated with the autophagy pathway showed coordinated transcriptional upregulation (Table S2) and SE splicing patterns in the transcriptome data and SE splicing events (Figure 6C–F); in contrast, Golgi transport-related genes exhibited complex splicing profiles (MXE+SE), with *rint1* showing transcriptional upregulation and *gopc* undergoing transcription-independent splicing reprogramming. The STEM analysis results revealed a critical dissociation between splicing dynamics and transcriptional expression, which underscored the temporal post-transcriptional regulation: while *gopc* showed no significant change in overall mRNA abundance throughout infection, *rint1* exhibited sustained transcriptional up-regulation. The autophagy genes (*pten*, *tsc2*, *vmp1*, *nt5c2b*) displayed fluctuating expression without a coherent clustering trend (Figure S2). These results suggest that the host utilized distinct regulatory strategies: the primary regulatory mechanism for *gopc* appeared to be at the splicing level, fine-tuning protein function without altering transcript quantity. Furthermore, in the WGCNA co-expression network of DAS genes, *gopc* shared a direct, exclusive connection with *rnf5*, an E3 ubiquitin ligase implicated as a negative regulator of autophagy [90,91,92,93], while other hub genes such as *rimoc1* and *golga4* are also associated with Golgi dynamics and membrane trafficking [52]. These associations suggest that *gopc* may occupy a key position within a gene regulatory module involved in vesicular transport and protein homeostasis.

Mutually exclusive exon (MXE) alternative splicing events can generate protein isoforms with distinct or even antagonistic functional domains [94]. For example, the Drosophila *mhc* (myosin heavy chain) gene utilizes this mechanism to produce diverse muscle myosins for regulating contraction. In contrast, skipped exon (SE) events primarily expand protein diversity via exon skipping, often leading to functional attenuation or structural disruption [94,95]. In our study, the Golgi vesicle transport related genes showed complex splicing patterns: the splicing maps of *gopc* and *rint1* each contained one MXE event, along with a relatively greater number of SE events (Figure 6A,B); in contrast, the splicing profiles of the four autophagy-related genes featured only a limited number of SE events (Figure 6C–F). However, according to records in the NCBI RefSeq database, MXE splicing events have not been reported for these two genes in humans, zebrafish (*Danio rerio*) or *L. crocea* [96,97,98,99,100,101]. The human *gopc* canonical isoform (RefSeq accession: NM_001017408.3) has been intensively studied for its involvement in regulation of Cystic Fibrosis Transmembrane conductance Regulator (CFTR), frizzled receptors, Epidermal Growth Factor Receptor (EGFR) trafficking, autophagy, or Golgi-endosome transport and synaptic vesicle recycling [102,103,104,105,106,107,108,109,110], and the splicing events of human *gopc* (SE, A3SS, RI type) are predicted to lead to alternation of dimerization or binding partners, and reduced interaction with PDZ ligands [96]. Recent studies have shown that siRNA-mediated knockdown of porcine *gopc* promotes the replication of the Classical Swine Fever Virus (CSFV) in Porcine Kidney-15 (PK-15) cells [111], suggesting that *gopc* may play a regulatory role in viral replication processes. Canonical human *rint1* (RefSeq accession: NM_001346603.2) is involved in the regulation of cell cycle, interaction with the ER tethering and SNARE (Soluble NSF Attachment Protein Receptor) complex, membrane trafficking and lipid metabolism, and its mutants could lead to liver disease by disrupting ER-Golgi transport, activating the unfolded protein response (UPR), and impairing autophagy [112], while the functions of the alternative splicing isoforms of this gene remain to be elucidated. The newly identified MXE splicing events of aquatic *gopc* and *rint1* homologs have not been clarified, this study could thus provide new insights into how these genes contribute to cellular responses during viral infections. Futher, the “SE only” autophagy-related genes may hierarchically influence cell fate via alternative splicing and translation [113,114,115,116], suggested distinct functional adaptations tailored to specific cellular demands during viral replication. These splicing alterations may serve to attenuate the efficiency of ER-Golgi trafficking, thereby promoting the accumulation of viral cargo within vesicles destined for non-lytic egress. Although *rint1* belongs to the significantly associated ME4 module in WGCNA (Figure 4F) and exhibits an MXE event, its failure to meet the network construction threshold suggests its regulatory role may differ from that of the central hub genes.

Overall, our findings suggest that megalocytivirus strain FD201807 may utilize host autophagy and Golgi vesicle transport pathways to support a non-lytic mode of dissemination during early infection in large yellow croaker cells. These same pathways are integral to the host innate immune system and play important roles in intracellular pathogen clearance [117,118]. The accumulation of capsid-deficient viral intermediates within intracellular vesicles at 48 hpi coincides with the first peak of viral genome copies and the exclusive detection of DAS gene enrichment (Figure 1C,D, Figure 2A and Figure 5C,D), implicating post-transcriptional regulation in this early phase. However, this non-lytic state is transient, as extensive CPEs emerge in later stages, indicating a switch to conventional lytic release (Figure 1B). Temporal analysis of viral load reveals a biphasic replication pattern: genome copies peak at 48 hpi, decline to a minimum at 120 hpi—paralleling the dynamic evolution of DEG abundance—and then rise again to a higher second peak at 144 hpi (Figure 2A). This rebound suggests that early non-lytic spread may enable broader cellular dissemination through vesicle-mediated transport, but the concurrent activation of host innate defenses, particularly autophagy, possibly leads to substantial loss of viral genomes during transmission, thereby contributing to the decline in viral load observed between 48 and 120 hpi (Figure 2A). Throughout the infection course, autophagy-related genes remain transcriptionally up-regulated (Figure S2), reflecting sustained activation of host defense responses. The final decline in viral genome copies coincides with extensive host cell lysis, likely reflecting exhaustion of viable cellular substrates for viral replication rather than active viral clearance.

We acknowledge several important limitations in the current study. First, the experimental design relies on an in vitro cell line model, which may not fully replicate the complex physiological environment and tissue-specific responses observed in vivo. Second, while the transcriptomic and WGCNA analyses provide robust predictive networks, these findings are currently based on computational inference and lack functional biological validation. In addition, although the multi-omics analyses revealed high inter-replicate consistency, the small sample size (*n* = 3) may constrain the statistical power to detect subtle expression changes. To address these limitations, future investigations will prioritize the application of functional genomic technologies, including gene knockdown, overexpression, and targeted genome editing, to verify the regulatory roles of key nodes. These mechanistic assays will ultimately inform the understanding of how viruses interface with host membrane trafficking machinery in the innate defense mechanisms of aquatic animals.

## 5. Conclusions

This study shows that megalocytivirus infection in large yellow croaker cells is accompanied by extensive changes in host alternative splicing, occurring concurrently with transcriptional alterations in autophagy and Golgi vesicle transport pathways, which are key components of animal innate immunity. The limited correlation between gene expression and splicing patterns, particularly the virus-associated occurrence of novel MXE events in *gopc* and *rint1*, suggests that alternative splicing contributes to the host innate response. Within the co-expression network of DAS genes, *gopc* exhibits a specific association with genes involved in ubiquitin-mediated autophagy regulation, implicating a potential role for isoform variation in vesicle-related processes during infection. The temporal alignment of capsid-deficient viral intermediates with non-lytic dissemination and a transient reduction in viral genome abundance implies that early engagement of intracellular trafficking and autophagy pathways associated with diminished viral levels, preceding the subsequent viral rebound. Collectively, these findings suggest a model wherein the virus may exploit host splicing and vesicle trafficking pathways to facilitate non-lytic egress. While these observations are currently correlative, they underscore the need for future functional studies to definitively elucidate the causal mechanisms of this potential viral hijacking strategy. Taken together, this work advances our understanding of the molecular basis of antiviral defense, which is fundamental to addressing iridovirus challenges in aquaculture.

## Figures and Tables

**Table 1:** Experimental sampling schedule. All groups originated from the same batch of infected YCE1 cells and were allocated into different groups for time-course analysis. The timing of sample collection varied depending on the requirements of each assay, as outlined below.

Sampling Time [hpi] ^1^	EM ^2^	MC ^3^	TR ^4^
24	+	+	+
48	+	+	+
72		+	
96	+	+	+
120		+	
144	+	+	+
168		+	

^1^ hpi: hours post-infection. ^2^ EM: electron microscopy. ^3^ MC: major capsid protein (*mcp*) gene copies measurement. ^4^ TR: transcriptomic analysis.

**Table 2:** The PCR primers use in this study.

Primer Name	Forward Sequence (5′-3′)	Reverse Sequence (5′-3′)	Amplicon Size [bp]	Accession Number	Purpose
*Q-MCP F/R*	CGGTATCACCAACGGTCAGACTATG	GGCAGAGACACGGTAGGCAATG	105	KY765672.1	Megalocytivirus *mcp* gene quantification
*β-actin-F/R*	GACCTGACAGACTACCTCATG	AGTTGAAGGTGGTCTCGTGGA	292	XM_027284923.1	Internal control
*psap-F/R*	GTGCCATCTGCGAGTTTGTG	GGTCCTTACACTGGCCCTTC	135	XM_027276328.1	Validation of transcriptomic temporal profiles
*rpl18a-F/R*	CTCGTGCTCACTCCATCCAG	GTGTTCGGTCTCTTGGTGGT	111	XM_027272485.1	Validation of transcriptomic temporal profiles
*LOC104920700-F/R*	ATCTGGAGGAGGATCACGCT	CTCTGCCAAGCCATCTTCCA	168	XM_010733046.3	Validation of transcriptomic temporal profiles
*map1lc3a-F/R*	GTCCGACAGACCCTTCAAACAG	CTCTCCCTTATACCGCTCAATGATG	151	XM_027279266.1	Validation of transcriptomic temporal profiles

## Data Availability

The data that support the findings of this study are available from the corresponding author upon reasonable request.

## Supplementary Materials

The following supporting information can be downloaded at: https://www.mdpi.com/article/10.3390/ani16081259/s1, Figure S1: Validation of RNA-sequencing (RNA-seq) reliability by comparing expression trends with qRT-PCR; Figure S2: Short time-series expression miner (STEM) analysis of temporal expression profiles for key genes identified by Gene Ontology (GO) and Kyoto Encyclopedia of Genes and Genomes (KEGG) enrichment analyses; Figure S3: Summary of the skipped exon (SE) DAS events for the six candidate genes; Table S1: The genes used in short time series expression miner (STEM) analysis; Table S2: Differentially expressed genes (DEGs) relevant to autophagy; Table S3: Differential alternative splicing (DAS) genes relevant to vesicle transport and autophagy; Table S4: Genes involved in the co-expression networks.

## Abbreviations

The following abbreviations are used in this manuscript:

CPEs	cytopathic effects
CSFV	Classical Swine Fever Virus
DAS	differential alternative splicing
ISKNV	infectious spleen and kidney necrosis virus
MCP	major capsid protein
MXE	mutually exclusive exon
MVB	multivesicular bodies
RGNNV	red-spotted grouper nervous necrosis virus
RPKM	Reads per Kilobase per Million Reads
rMATS	replicate Multivariate Analysis of Transcript Splicing
RSIV	red seabream iridovirus
SNARE	Soluble NSF Attachment Protein Receptor
SE	skipped exon
SGIV	Singapore grouper iridovirus
STEM	short time-series expression miner
YCE1	Yellow Croaker Embryonic type-I cell line
