# Supplementary material for: Transcriptional and Alternative Splicing Regulation of Autophagy and Vesicle Transport Pathways in Large Yellow Croaker Cells During Megalocytivirus Infection
Source: Animals (Basel). 2026 Apr 20;16(8):1259. doi: 10.3390/ani16081259 (PMC13113295; doi:10.3390/ani16081259)
Supplement: Supplementary file 1 [file animals-16-01259-s001.zip › Table S4. Genes involved in the co-expression networks.pdf]

Table S4. Genes involved in the co-expression networks

Group	Gene Symbol	Full Description	Note
DEGs	*mapk9*[a]	Mitogen-activated protein kinase 9	Hub Gene
	map1lc3a[a]	Microtubule associated protein 1 light chain 3 alpha	Hub Gene
	rraga	Ras related gtp binding a	
	rragca (A)	Ras related gtp binding c a (isoform 1)	
	rragca	Ras related gtp binding c a (isoform 2)	
	tp53inp2b	Tumor protein p53 inducible nuclear protein 2b	
	wipi1 (C)	Wd repeat domain, phosphoinositide interacting 1c	
	adgrd1	Adhesion gpcr d1	
	wipi1 (A)	Wd repeat domain, phosphoinositide interacting 1a	
	bnip4	Bcl2/adenovirus e1b 19kda interacting protein 4	
	wipi1 (B)	Wd repeat domain, phosphoinositide interacting 1b	
	eif2ak3	Eukaryotic translation initiation factor 2 alpha kinase 3	
	gabarapa	Gaba type a receptor associated protein a	
	dapk3	Death associated protein kinase 3	
	fancb	Fa complementation group b	
	ulk1a	Unc-51 like autophagy activating kinase 1a	
	khc	Kinesin heavy chain	
	atg7	Autophagy related 7	
	vamp8	Vesicle associated membrane protein 8	
	zfyve1	Zinc finger fyve-type containing 1	
	gabarapl2	Gaba type a receptor associated protein like 2	
	wipi1 (D)	Wd repeat domain, phosphoinositide interacting 1d	
	myo1b (A)	Myosin ib	
	dapk2	Death associated protein kinase 2	
	retreg1	Reticulophagy regulator 1	
	ctsl	Cathepsin l	
DAS genes	*rimoc1*[a]	Rab7a interacting mon1-ccz1 complex subunit 1	Hub Gene
	rnf5[a]	Ring finger protein 5	Hub Gene
	golga4[a]	Golgin a4	Hub Gene
	gopc[b]	Golgi-associated pdz and coiled-coil motif containing	Enriched
	rorabl1	Ras-related protein orab-1-like	
	mpv17l2	Mpv17 mitochondrial inner membrane protein like 2	
	grik1a	Glutamate receptor, ionotropic, kainate 1a	
	nrgnb	Neurogranin b	
	ank2b	Ankyrin 2b, neuronal	
	loc104931761	Uncharacterized loc104931761	
	pum2	Pumilio rna-binding family member 2	
	cadm1b	Cell adhesion molecule 1b	
	arhgef1	Rho guanine nucleotide exchange factor 1	
	dennd5b	Denn/madd domain containing 5b	
	znf135	Zinc finger protein 135	
	klf5l	Kruppel like factor 5 like	

nbr1b	Nbr1 autophagy cargo receptor b
stx18	Syntaxin 18
dync1li2	Dynein cytoplasmic 1 light intermediate chain 2
rab6a	Member of ras oncogene family 6a
loc104923982	Uncharacterized loc104923982
arfgap1	Adp-ribosylation factor gtpase activating protein 1
vav2	Vav 2 guanine nucleotide exchange factor
afap1l1b	Actin filament associated protein 1-like 1b
rictor	Rapamycin-insensitive companion of mtor

1. [a] Hub genes identified based on network connectivity (*mapk9*, *map1lc3a* for DEGs; *rimoc1*, *rnf5*, *golga4* for DAS).

2. [b] Functionally enriched gene (*gopc*).

3. Gene symbols with parenthetical letters (e.g., wipi1 (D)) indicate specific isoforms shown in the network diagrams.
