# Supplementary material for: Transcriptional and Alternative Splicing Regulation of Autophagy and Vesicle Transport Pathways in Large Yellow Croaker Cells During Megalocytivirus Infection
Source: Animals (Basel). 2026 Apr 20;16(8):1259. doi: 10.3390/ani16081259 (PMC13113295; doi:10.3390/ani16081259)
Supplement: Supplementary file 1 [file animals-16-01259-s001.zip › Supplementary figure captions.pdf]

Supplementary Figure captions

Figure S1. Validation of RNA-sequencing (RNA-seq) reliability by comparing expression trends with qRT-PCR. Temporal expression patterns of four candidate differentially expressed genes (DEGs) were compared between RNA-seq and quantitative real-time PCR (qRT-PCR) in FD201807-infected YCE1 cells. The relative expression levels of (**A**) *psap*, (**B**) *rpl18a*, (C) *LOC104920700*, and (**D**) *map1lc3a* showed consistent trends between the two methods. The results of qRT-PCR are shown as $2^{-\Delta\Delta CT}$ values, using *β*-actin as the reference gene (*n* = 3); the results of RNA-seq are shown as mean RPKM values ± SD (*n* = 3).

Figure S2. Short time-series expression miner (STEM) analysis of temporal expression profiles for key genes identified by Gene Ontology (GO) and Kyoto Encyclopedia of Genes and Genomes (KEGG) enrichment analyses. (**A**) Significant up-regulation trend observed in 8 out of 15 key genes associated with autophagy and Golgi vesicle transport. (**B**) Heatmap illustrating the expression levels of these 8 genes across different time points, corresponding to the profile shown in panel (**A**).

Figure S3. Summary of the skipped exon (SE) DAS events for the six candidate genes. This figure specifically highlights the SE-type differential alternative splicing (DAS) events. Sashimi plots comparing splicing patterns between the control (red) and FD201807-infected (yellow) groups are shown for (A) *gopc*, (B) *rint1*, (C) *tsc2*, (D) *vmp1*, (E) *pten*, and (F) *nt5c2b*. The x-axis represents genomic coordinates, and the y-axis shows normalized RPKM values. Arc heights correspond to splice junction read counts (numerically labeled), while IncLevel indicates the normalized exon inclusion ratio. For the comprehensive splicing profiles of each gene, please refer to Figure 6. Full gene descriptions are listed in Table S1.
