# Supplementary material for: Transcriptional and Alternative Splicing Regulation of Autophagy and Vesicle Transport Pathways in Large Yellow Croaker Cells During Megalocytivirus Infection
Source: Animals (Basel). 2026 Apr 20;16(8):1259. doi: 10.3390/ani16081259 (PMC13113295; doi:10.3390/ani16081259)
Supplement: Supplementary file 1 [file animals-16-01259-s001.zip › Table S1. The genes used in shot time series expression miner (STEM) analysis.pdf]

Table S1. The genes used in short time series expression miner (STEM) analysis

Gene name	Accession Number	Full description	DEG	DAS	GO	GO term	KEGG	KEGG pathway	Long-term trend
LOC104934108	XM_010749693.3/ XM_027291213.1 (Common region)	Atypical chemkine receptor 3 (LOC104934108)	+	-	-	-	-	-	-
frem1b	XM_027290530.1/ XM_027290531.1/ XM_027290529.1/ XM_027290533.1 (Common region)	Fras1 related extracellular matrix 1b	+	-	-	-	-	-	-
map1lc3a	XM_027279266.1/ XM_027279267.1 (Common region)	microtubule-associated protein 1 light chain 3 alpha	+	-	+	GO: 0006914	-	-	Up-regulated
gabarapa	XM_010737173.3	GABA(A) receptor-associated protein a	+	-	+	GO: 0006914	+	lco04140	Up-regulated
gabarapl2	XM_010749336.3	GABA(A) receptor-associated protein like 2	+	-	+	GO: 0006914	+	lco04140	Up-regulated
wipi1 (a)	XM_019268246.2	WD repeat domain, phosphoinositide interacting 1	+	-	+	GO: 0006914	+	lco04140	Up-regulated
wipi1 (b)	XM_019268245.2	WD repeat domain, phosphoinositide interacting 1	+	-	+	GO: 0006914	+	lco04140	Up-regulated
wipi1 (c)	XM_010736306.3	WD repeat domain phosphoinositide-interacting protein 1	+	-	+	GO: 0006914	+	lco04140	Up-regulated
gopc	XM_010756080.3	Golgi-associated PDZ and coiled-coil motif containing	-	+	+	GO: 0048193	-	-	-
rint1	XM_027289135.1	RAD50 interactor 1	-	+	+	GO: 0048193	-	-	Up-regulated
pten	XM_010744227.3	Phosphatidylinositol 3,4,5-trisphosphate 3-phosphatase and dual-specificity protein phosphatase	-	+	-	-	+	lco04140	-
tsc2	XM_019260939.2	TSC complex subunit 2	-	+	-	-	+	lco04140	-
nt5c2b	XM_019260180.2/ XM_027282763.1 (Common region)	5'-nucleotidase, cytosolic IIb	-	+	-	-	+	lco04140	-
vmp1	XM_019255433.2	Vacuole membrane protein 1	-	+	-	-	+	lco04140	-
