# Supplementary material for: Transcriptional and Alternative Splicing Regulation of Autophagy and Vesicle Transport Pathways in Large Yellow Croaker Cells During Megalocytivirus Infection
Source: Animals (Basel). 2026 Apr 20;16(8):1259. doi: 10.3390/ani16081259 (PMC13113295; doi:10.3390/ani16081259)
Supplement: Supplementary file 1 [file animals-16-01259-s001.zip › Table S2. Differentially expressed genes (DEGs) relevant to autophagy.pdf]

Table S2. Differentially expressed genes (DEGs) relevant to autophagy

Gene name	GO	Group	GO term	KEGG	Group	KEGG pathway	Expression trend
mtcl3	+	FD_24h vs. Ctrl_24h<br>FD_96h vs. FD_24h	GO:0006914<br>GO:0010506	-	-	-	Down-regulated
klhl21	+	FD_24h vs. Ctrl_24h	GO:0006914<br>GO:0010506	-	-	-	Down-regulated
retreg1	+	FD_24h vs. Ctrl_24h<br>FD_96h vs. FD_24h	GO:0006914	-	-	-	Down-regulated
map1lc3a	+	FD_96h vs. FD_24h	GO:0006914	-	-	-	Up-regulated
gabarapa	+	FD_96h vs. FD_24h	GO:0006914	+	FD_96h vs. FD_24h<br>FD_144h vs. FD_24h	lco04136<br>lco04140	Up-regulated
gabarapl2	+	FD_96h vs. FD_24h	GO:0006914	+	FD_96h vs. Ctrl_96h<br>FD_96h vs. FD_24h<br>FD_144h vs. FD_24h<br>FD_144h vs. FD_48h	lco04136<br>lco04140	Up-regulated
wipi1a	+	FD_96h vs. FD_24h	GO:0006914	+	FD_96h vs. FD_24h<br>FD_96h vs. FD_48h<br>FD_144h vs. FD_24h<br>FD_144h vs. FD_48h	lco04136<br>lco04140	Up-regulated
wipi1b	+	FD_96h vs. FD_24h	GO:0006914	+	FD_96h vs. FD_24h<br>FD_96h vs. FD_48h<br>FD_144h vs. FD_24h<br>FD_144h vs. FD_48h	lco04136<br>lco04140	Up-regulated
wipi1c	+	FD_96h vs. FD_24h	GO:0006914	+	FD_96h vs. FD_24h<br>FD_144h vs. FD_24h<br>FD_144h vs. FD_48h	lco04136<br>lco04140	Up-regulated
adgrd1	-	-	-	+	FD_96h vs. FD_24h<br>FD_96h vs. FD_48h<br>FD_144h vs. FD_24h<br>FD_144h vs. FD_48h	lco04140	Up-regulated
atg16l2	-	-	-	+	FD_144h vs. FD_24h	lco04136<br>lco04140	Up-regulated
atg7	-	-	-	+	FD_144h vs. FD_24h	lco04136<br>lco04140	Up-regulated
bcl2	-	-	-	+	FD_96h vs. Ctrl_96h<br>FD_96h vs. FD_24h<br>FD_96h vs. FD_48h<br>FD_144h vs. FD_24h<br>FD_144h vs. FD_48h	lco04136<br>lco04140	Up-regulated
bnip4	-	-	-	+	FD_96h vs. Ctrl_96h<br>FD_96h vs. FD_24h<br>FD_96h vs. FD_48h<br>FD_144h vs. FD_24h<br>FD_144h vs. FD_48h	lco04136<br>lco04140	Up-regulated
ctsd	-	-	-	+	FD_144h vs. FD_24h	lco04140	Up-regulated
ctsl	-	-	-	+	FD_144h vs. FD_24h	lco04140	Up-regulated
dapk2	-	-	-	+	FD_144h vs. FD_24h	lco04140	Up-regulated
dapk3	-	-	-	+	FD_96h vs. Ctrl_96h<br>FD_96h vs. FD_24h	lco04136<br>lco04140	Up-regulated

					FD_96h vs. FD_48h<br>FD_144h vs. FD_24h<br>FD_144h vs. FD_48h		
ddit4	-	-	-	+	FD_144h vs. FD_24h	lco04140	Up-regulated
eif2ak3	-	-	-	+	FD_144h vs. FD_24h	lco04140	Up-regulated
ern2	-	-	-	+	FD_96h vs. FD_48h<br>FD_144h vs. FD_48h	lco04140	Up-regulated
fancb	-	-	-	+	FD_144h vs. FD_24h<br>FD_144h vs. FD_48h	lco04140	Up-regulated
khc	-	-	-	+	FD_144h vs. FD_48h	lco04140	Up-regulated
lamp1a	-	-	-	+	FD_96h vs. FD_24h<br>FD_144h vs. FD_24h<br>FD_144h vs. FD_48h	lco04140	Up-regulated
mapk9	-	-	-	+	FD_96h vs. FD_24h<br>FD_144h vs. FD_24h<br>FD_144h vs. FD_48h	lco04140	Up-regulated
mrasa	-	-	-	+	FD_144h vs. FD_24h<br>FD_144h vs. FD_48h	lco04140	Up-regulated
napsa	-	-	-	+	FD_144h vs. FD_24h	lco04140	Up-regulated
rheb	-	-	-	+	FD_144h vs. FD_48h	lco04140	Up-regulated
rraga	-	-	-	+	FD_144h vs. FD_24h	lco04140	Up-regulated
rragca	-	-	-	+	FD_96h vs. FD_24h<br>FD_144h vs. FD_24h<br>FD_144h vs. FD_48h	lco04140	Up-regulated
rragcb	-	-	-	+	FD_144h vs. FD_24h	lco04136	Up-regulated
tgfbr2l	-	-	-	+	FD_96h vs. FD_24h	lco04136	Up-regulated
tp53inp2b	-	-	-	+	FD_96h vs. FD_24h<br>FD_96h vs. FD_48h<br>FD_144h vs. FD_24h<br>FD_144h vs. FD_48h	lco04140	Up-regulated
ulk1a	-	-	-	+	FD_96h vs. FD_24h<br>FD_96h vs. FD_48h<br>FD_144h vs. FD_24h<br>FD_144h vs. FD_48h	lco04136<br>lco04140	Up-regulated
ulk1b	-	-	-	+	FD_96h vs. FD_24h	lco04140	Up-regulated
ulk2	-	-	-	+	FD_144h vs. FD_24h<br>FD_144h vs. FD_48h	lco04136<br>lco04140	Up-regulated
vamp8	-	-	-	+	FD_144h vs. FD_48h	lco04140	Up-regulated
zfyve1	-	-	-	+	FD_144h vs. FD_24h	lco04140	Up-regulated
