# Supplementary material for: Transcriptional and Alternative Splicing Regulation of Autophagy and Vesicle Transport Pathways in Large Yellow Croaker Cells During Megalocytivirus Infection
Source: Animals (Basel). 2026 Apr 20;16(8):1259. doi: 10.3390/ani16081259 (PMC13113295; doi:10.3390/ani16081259)
Supplement: Supplementary file 1 [file animals-16-01259-s001.zip › Table S3. Differential alternative splicing (DAS) genes relevant to vesicle transport and autophagy.pdf]

Table S3. Differential alternative splicing (DAS) genes relevant to vesicle transport and autophagy

Gene name	GO	Group	GO term	KEGG	Group	KEGG pathway	MXE	SE
gopc	+	FD_48h vs. Ctrl_48h	GO:0048193	-	-	-	1	6
rint1	+	FD_48h vs. Ctrl_48h	GO:0048193	-	-	-	1	5
tsc2	-	-	-	+	FD_48h vs. Ctrl_48h	lco04140	-	3
vmp1	-	-	-	+	FD_48h vs. Ctrl_48h	lco04140	-	3
pten	-	-	-	+	FD_48h vs. Ctrl_48h	lco04140	-	2
nt5c2b	-	-	-	+	FD_48h vs. Ctrl_48h	lco04140	-	1
