# Supplementary figures and images for: Transcriptional and Alternative Splicing Regulation of Autophagy and Vesicle Transport Pathways in Large Yellow Croaker Cells During Megalocytivirus Infection
Source: Animals (Basel). 2026 Apr 20;16(8):1259. doi: 10.3390/ani16081259 (PMC13113295; doi:10.3390/ani16081259)

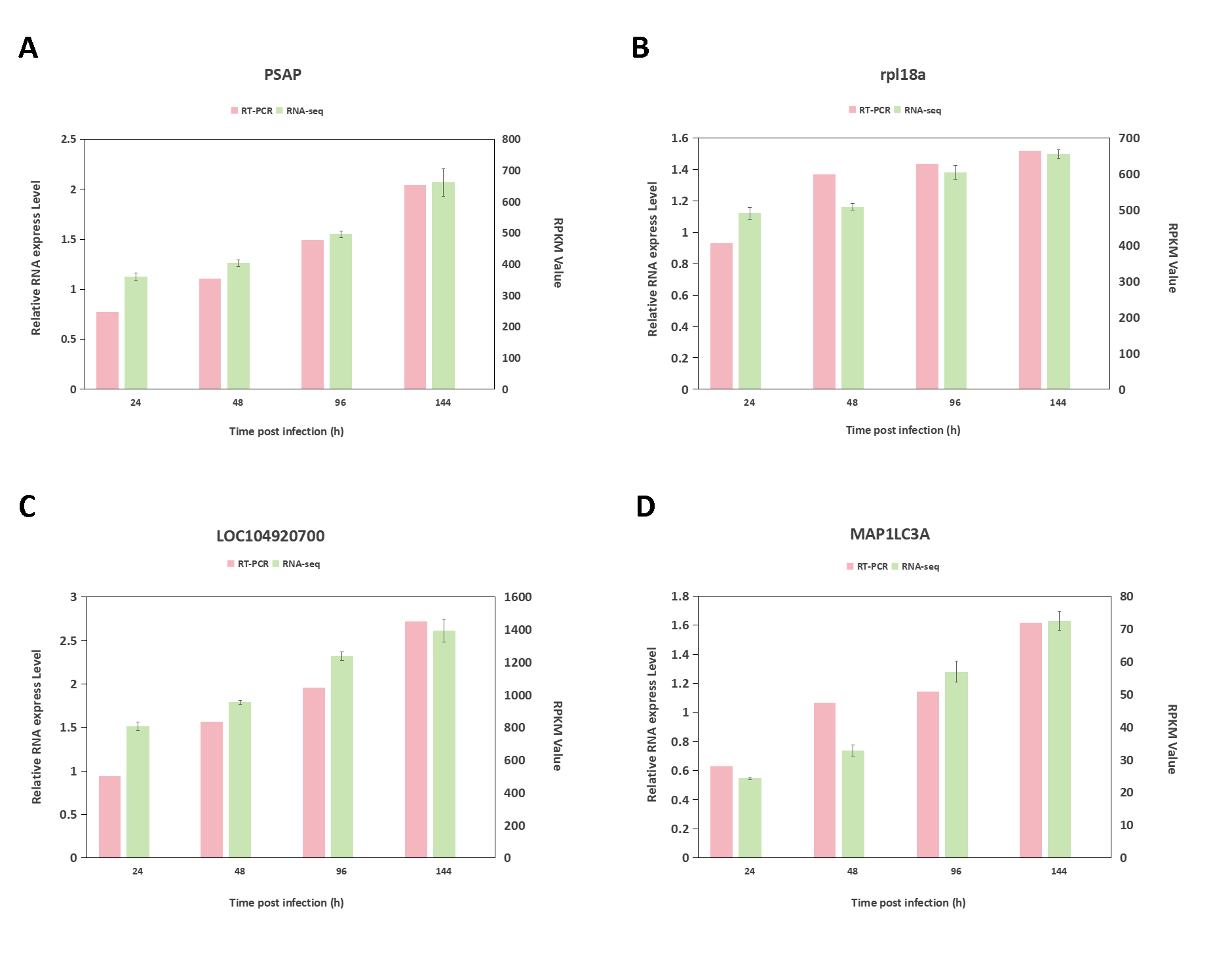

Supplement: Supplementary file 1 [file animals-16-01259-s001.zip › Figure S1.png]

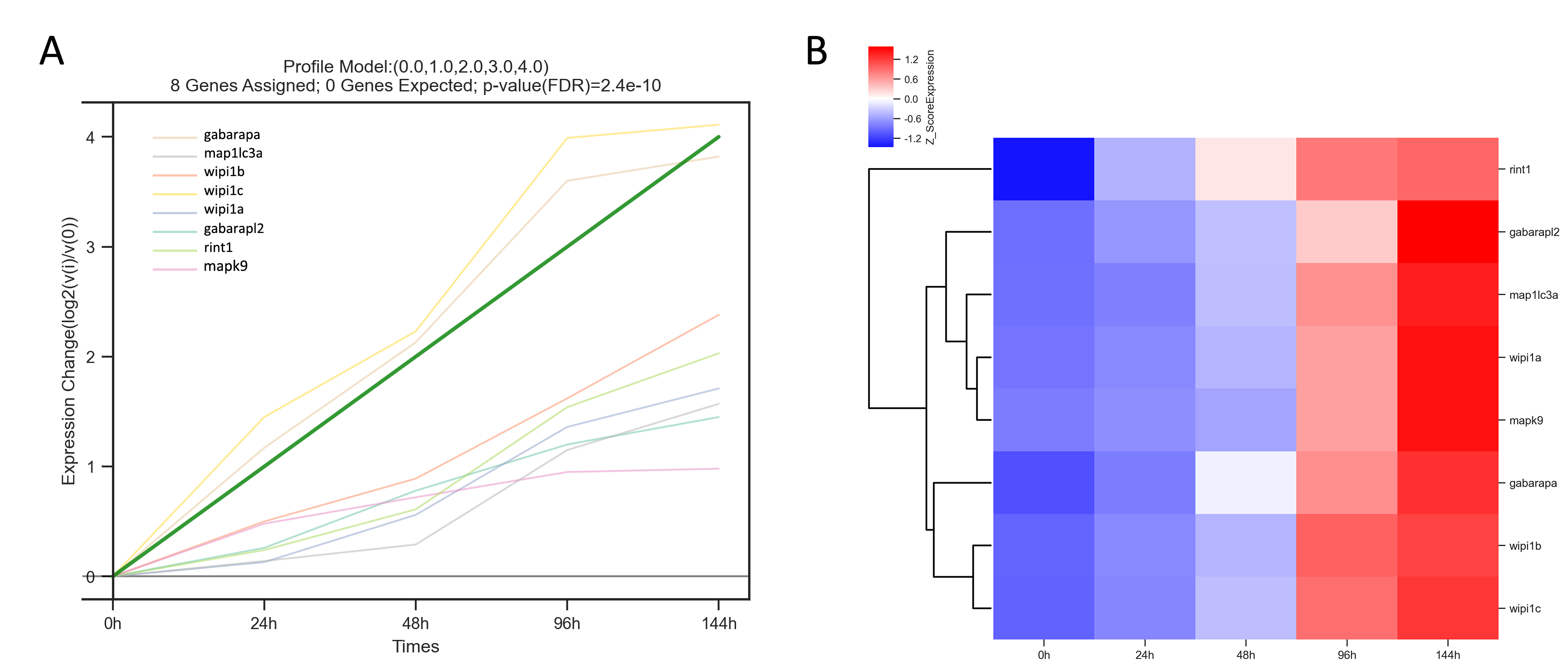

Supplement: Supplementary file 1 [file animals-16-01259-s001.zip › Figure S2.png]

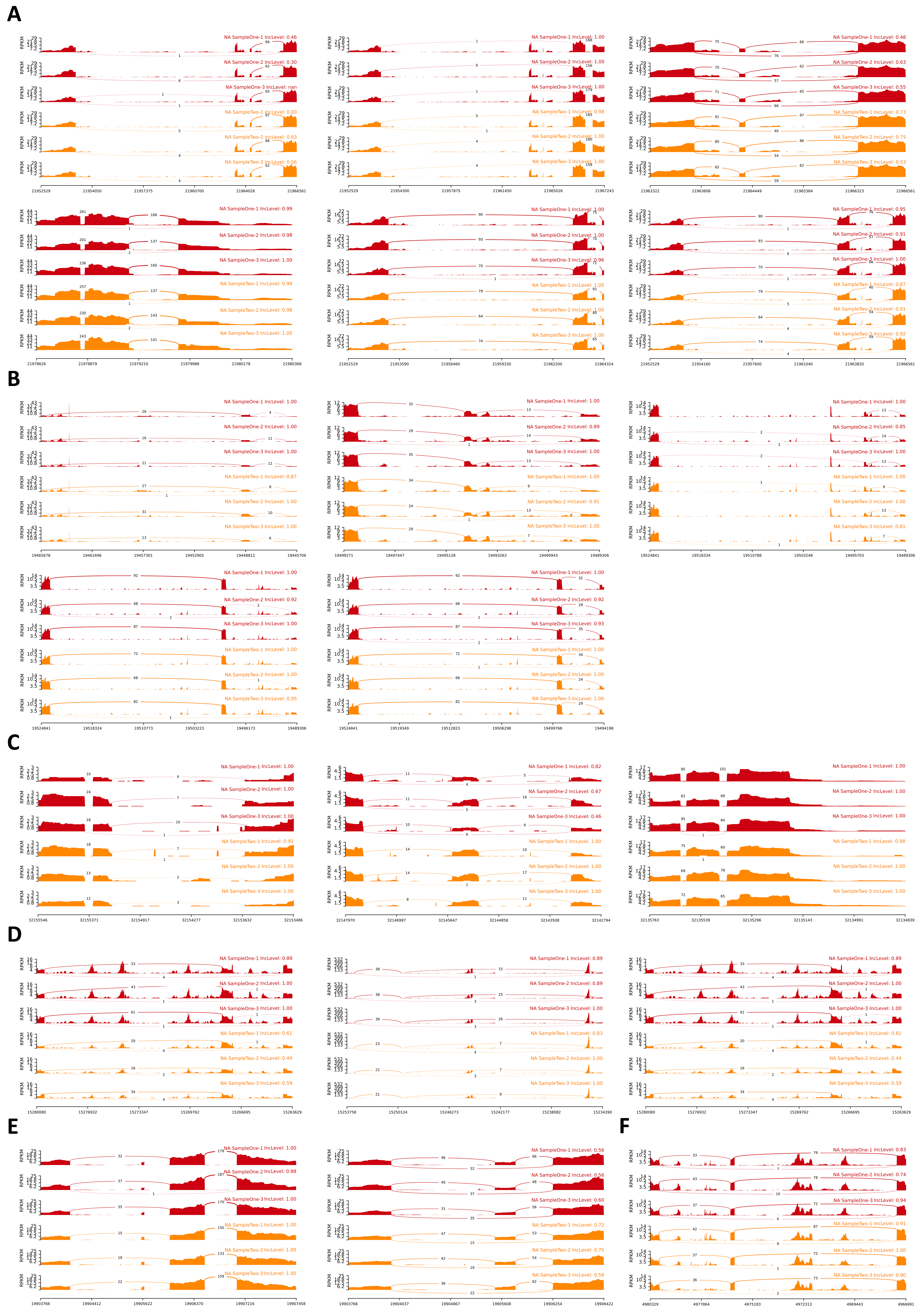

Supplement: Supplementary file 1 [file animals-16-01259-s001.zip › Figure S3.png]
